# Heterogeneity of circulating CD4^+^CD8^+^ double-positive T cells characterized by scRNA-seq analysis and trajectory inference

**DOI:** 10.1038/s41598-022-18340-3

**Published:** 2022-08-18

**Authors:** Sung Min Choi, Hi Jung Park, Eun A Choi, Kyeong Cheon Jung, Jae Il Lee

**Affiliations:** 1 Graduate Course of Translational Medicine, Seoul National University College of Medicine, Seoul, 03080 Republic of Korea; 2 Transplantation Research Institute, Seoul National University College of Medicine, Seoul, 03080 Republic of Korea; 3 Department of Pathology, Seoul National University College of Medicine, Seoul, 03080 Republic of Korea; 4 Integrated Major in Innovative Medical Science, Seoul National University Graduate School, Seoul, 03080 Republic of Korea; 5 Department of Medicine, Seoul National University College of Medicine, Seoul, 03080 Republic of Korea

**Keywords:** Computational biology and bioinformatics, Immunology

## Abstract

The frequency of CD4^+^CD8^+^ double-positive (DP) T cells is highly associated with a variety of diseases. Recently, we used high-throughput single-cell RNA sequencing to show that circulating DP T cells in cynomolgus monkeys comprise nine heterogeneous populations. To better understand the characteristics of DP T cells, we analyzed 7601 cells from a rhesus monkey and detected 14,459 genes. Rhesus monkey DP T cells comprised heterogeneous populations (naïve, Treg-, Tfh-, CCR9^+^ Th-, Th17-, Th2-, Eomes^+^ Tr1-, CTL-, PLZF^+^ innate- and Eomes^+^ innate-like cells) with multiple potential functions. We also identified two new subsets using aggregated scRNA-seq datasets from the rhesus and the cynomolgus monkey: CCR9^+^ Th-like cells expressing *ICAM2* and *ITGA1*, and PLZF^+^ innate-like cells that display innate-associated gene signatures such as *ZBTB16*, *TYROBP*, *MAP3K8*, and *KLRB1*. Trajectory inference of cell differentiation status showed that most DP T cells in the rhesus monkey were found in the mid-to-late pseudotime, whereas DP T cells from the cynomolgus monkey were found in early pseudotime. This suggests that DP T cells in rhesus monkeys may exhibit more diverse differentiation states than those in cynomolgus monkeys. Thus, scRNA-seq and trajectory inference identified a more diverse subset of the circulating DP T cells than originally thought.

## Introduction

Single-cell RNA sequence (scRNA-seq) profiling has enabled high-resolution mapping of cellular heterogeneity, development, and activation status in diverse immune systems^1,2^. Furthermore, trajectory inference has radically enhanced scRNA-seq research by enabling the study of dynamic changes in gene expression^3^. This approach can achieve a more detailed single-cell level characterization of a limited T cell subset present in peripheral blood.

Recently, we reported that CD4^+^CD8^+^ double-positive (DP) T cells from cynomolgus monkeys comprise nine heterogeneous cell populations^4^. Notably, we found that in terms of the presence of a substantial number of the naïve DP T cells expressing CD8*αα*, along with high expression of *PECAM1*(CD31; a thymic emigrant marker), this population might be one of the mature T cell lineages of thymic origin^4,5^. In addition, identification of Eomes^+^ innate-like cells also has important implications for the diverse functions and roles of DP T cells. These findings demonstrate that peripheral DP T cells may have regulatory properties^6^, or follicular helper^7^ or innate and adaptive functions^8^. Thus, the abundance of DP T cells in the blood and/or target organs supports their involvement in pathological processes.

Like CD4^+^ or CD8^+^ single-positive T cells, the frequency of CD4^+^CD8^+^ DP T cells is readily observed in peripheral blood, but their composition, activation status, and immunological significance are poorly understood. Here, we used the scRNA-seq and trajectory platforms to analyze the characteristics of DP T cells from a rhesus monkey, and compare their transcriptomic profiles with those of cells from the cynomolgus monkey.

## Results

### scRNA-seq analysis of CD4^+^CD8^+^ DP T cells

**Figure 1:**
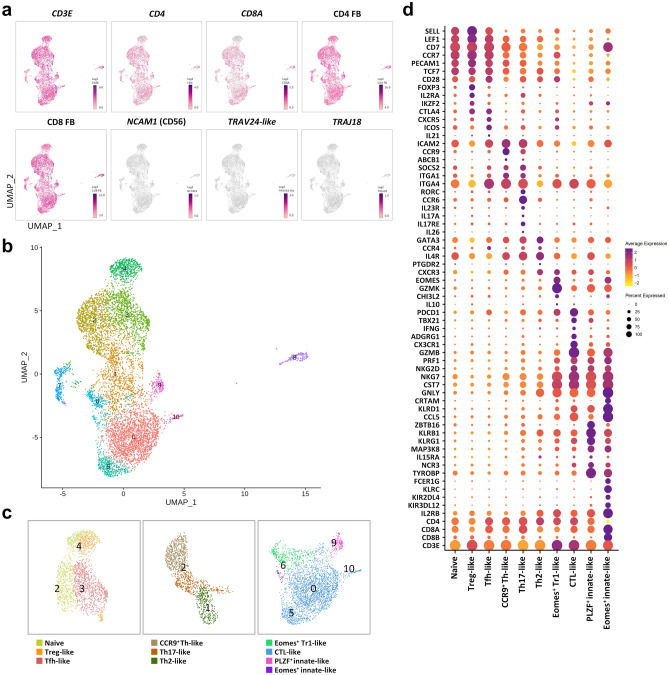
Single-cell RNA-seq analysis of CD4^+^CD8^+^ DP T cells from rhesus monkey. (**a**) UMAP of *CD3E*, *CD4*, *CD8A*, CD4 FB, CD8 FB, *NCAM1* (CD56), *TRAV24*-like (TCRVα24), and *TRAJ18* (TCRJα18) expression by CD4^+^CD8^+^ DP T cells. (**b**) UMAP representing the clusters of DP T cells from the rhesus monkey. The nearest neighbor algorithm on the Seurat analysis platform revealed 11 distinct clusters (numbered). After filtering, (**c**) ten cell types were defined according to expression of marker genes. The number in UMAP indicates the cluster number. Each color represents a defined DP T cell subset. (**d**) Dot plot showing expression of marker genes in DP T cell subsets. Color represents the average expression of the marker genes, and the dot size indicates the percentage of cells expressing the marker genes in the subsets.

To analyze circulating CD4^+^CD8^+^ DP T cells, we first separated peripheral DP T cells from a healthy adult rhesus monkey using MACS microbeads. Isolated DP T cells were labeled with CD4 and CD8α feature barcodes (FB), also known as an antibody-derived tag (ADT). We then ran the 10 × Genomics single-cell RNA sequencing. After filtering, 14,459 genes were detected and analyzed from 7601 cells. High expressions of *CD3E*, CD4 FB, and CD8 FB were identified in all clusters of sorted DP T cells. In contrast, the expression of *NCAM1* (CD56), *TRAV24* (TCRVα24-like), and *TRAJ18* (TCRjα18) associated with NK or NKT was very low or absent (Fig. 1a). That is, the analyzed cells are all CD4^+^CD8^+^ DP T cells.

### Heterogeneity within the CD4^+^CD8^+^ DP T cell population

The 11 clusters were identified using the nearest neighbor algorithm in the Seurat analysis platform (Fig. 1b). And then, ten cell types were classified based on differential expression and UMAP coordinates for specific marker genes (Fig. 1c and d, Supplementary Fig. S1a). We found that clusters (comprising parts of clusters 2, 3, and 4) of a substantial number of single cells shared naïve cell gene signatures such as *SELL, LEF1*, *CD7*, *CCR7*, *PECAM1*, and *TCF7*. To determine whether these cells differ from thymic immature DP T cells, we examined the expression of genes associated with stage markers. The immature DP stage markers *AQP3* and *SMPD3*^9^ were both absent (Supplementary Fig. S1b). We found that part of cluster 3 and cluster 4 can be defined as Treg-like cells based on high differential expression of regulatory T cell-associated gene signatures *FOXP3*, *IL2RA*, *CTLA4*, and *IKZF2*^10^. We also identified a cluster that could be defined as circulating follicular helper T-like cells. This cluster, the majority of cluster 3, showed high differential expression of genes *CXCR5*, *ICOS*, *PDCD1*, and *IL21*^11^, along with homing receptors *CCR7* and *SELL*^12^. In a part of cluster 2, we found a distinct subset showing high expression of *CCR9* and *ICAM2*. We also identified genes (*ITGA1*, *ABCB1*^13^, and *ITGA4*.) associated with blood CCR9^+^ Th cells. Thus, these cells were denoted as CCR9^+^ Th-like cells. Next, we identified differentially expressed genes by two clusters that showed gene signatures of helper T cell lineages. *RORC*, *CCR6*, *IL23R*, *IL17A*, and *IL17RE*^10^ were highly expressed in part of cluster 2. Part of cluster 1 exhibited high expression of Th2 cell-associated genes such as *GATA3*, *CCR4*, *IL4R*, and *PTGDR2*^10^. Considering these gene signatures, we defined some cells in clusters 1 and 2 as Th2-like cells and Th17-like cells, respectively. Cluster 6 showed genes signatures of Eomes^+^ type 1 regulatory T (Tr1)-like cells such as *EOMES*, *GZMK*, *CHI3L2*, *CST7*, and *NKG7*^14^, but the canonical Treg markers *IL2RA* and *FOXP3*^14^ were not identified. Given these findings, the majority of cells in cluster 6 were defined as Eomes^+^ Tr1-like cells. Next, we identified a large cluster (clusters 0, 5, and 10) in which a large number of single-cells shared similar gene markers associated with cytotoxic function, such as *GZMB*, *NKG7*, *CST7*, *CCL5*, *CX3CR1*, *KLRD1*, *PRF1*, *NKG2D*, and *ADGRG1*^15–18^. This cluster was defined as cytotoxic T lymphocyte (CTL)-like cells according to the differentially expressed gene signatures. Lastly, we found scRNA-seq data that could be divided into two distinct clusters (cluster 9 and part of cluster 6). Cluster 9 was characterized by exclusive and high expression of *ZBTB16*. In particular, this cluster also expressed high levels of *KLRB1, MAP3K8,* and *TYROBP,* which are known as innate-like markers^19–21^, along with *KLRG1* and *KLRD1.* By contrast, part of cluster 6, a very small subset, exhibited high expression of *EOMES*, suggesting a potential difference in function. Within this cluster, the killer cell-like receptor (KLR) family (e.g., *KLRC* and *KLRB1*)^22^ and killer cell immunoglobulin-like receptor (KIR) family genes (e.g., *KIRD2DL4* and *KIR3DL12*)^23^ were expressed exclusively. Additionally, *IL2RB*, *NCR3*, *FCER1G*, and *TYROBP*, all of which are associated with innate-like function^23^, were also highly expressed. Based on these gene signatures, we defined cluster 9 and the part of cluster 6 as PLZF^+^ innate- and Eomes^+^ innate-like cells, respectively.

### Analysis of aggregated scRNA-seq data from the rhesus and a cynomolgus monkey

**Figure 2:**
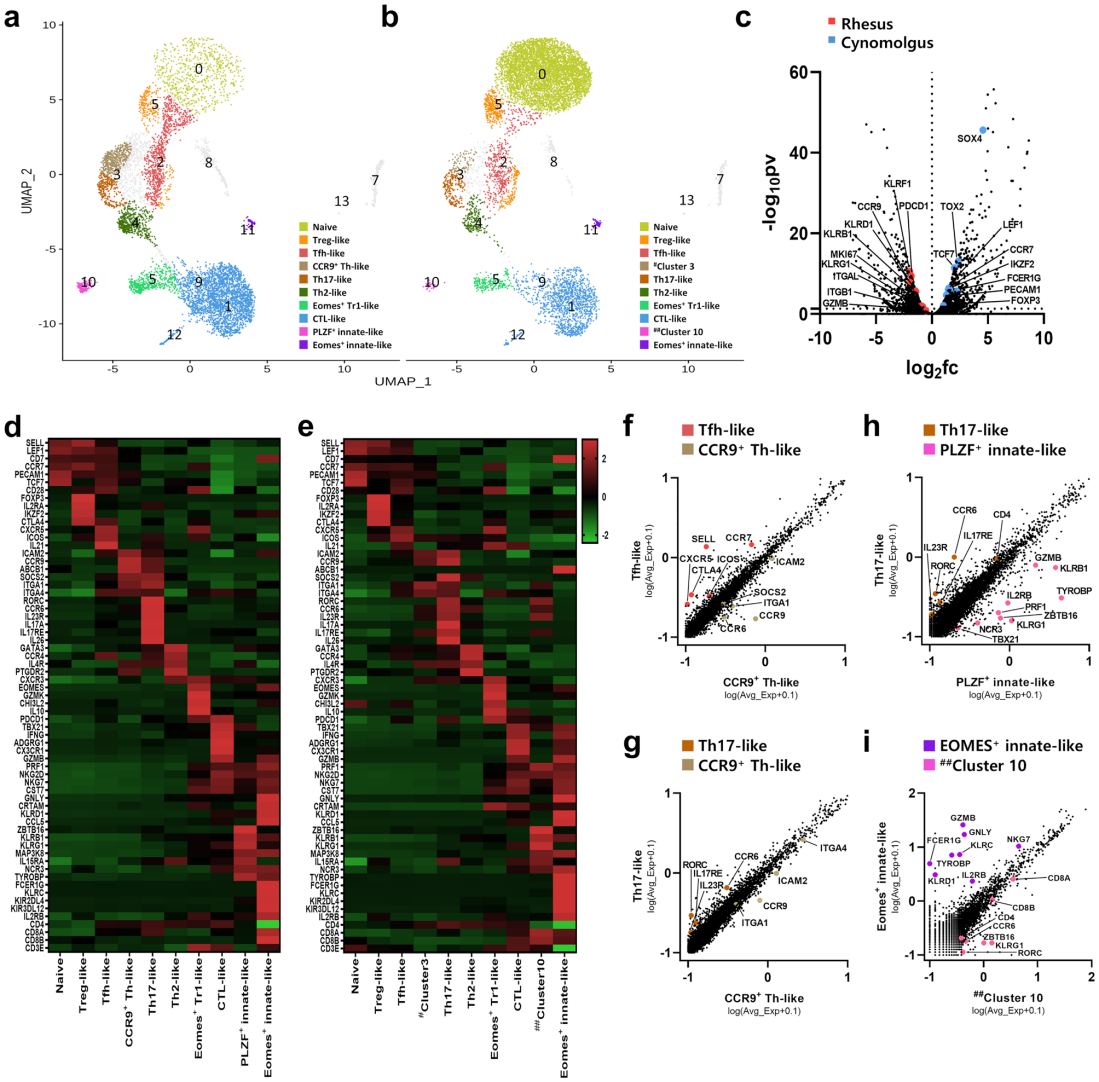
(**a, b**) Transcriptome-based clustering of an aggregated dataset for DP T cells from the rhesus and a cynomolgus monkey. For DP T cells from the cynomolgus monkey, data published by Choi et al. ^4^ were used. Fourteen distinct clusters (numbered) were identified among aggregated DP T cells from the two monkeys, and the UMAP is shown for each DP T cell from (**a**) the rhesus and (**b**) a cynomolgus monkey. Each color represents the defined DP T cell subset. (**c**) Volcano plot showing the log(fold change) and -log(*p*-values) for differentially expressed genes between DP T cells from the rhesus and the cynomolgus monkey. The dotted horizontal line represents a *P*-value of 0.05. (**d, e**) Heatmap showing relative expression of marker genes representing the cell types defined by the aggregated DP T cell data from (**d**) the rhesus and (**e**) a cynomolgus monkey; shown are the Z-scores obtained by differentially expressed gene analysis of all defined cell types. (**f–i**) Scatter plot of differentially expressed genes expressed by two DP T subtypes. (f) Tfh-like cells *vs.* CCR9^+^ Th-like in the rhesus monkey. (g) Th17-like cells *vs.* CCR9^+^ Th-like in the rhesus monkey. (h) Th17-like cells *vs.* PLZF^+^ innate-like in the rhesus monkey. (**i**) Eomes^+^ innate-like cells *vs.* ^##^cluster 10 (*ZBTB16*-expressing cells) in the cynomolgus monkey. All plots were generated using GraphPad Prism 8.0.2.

To investigate differences in DP T cell clustering by differentially expressed genes between the two monkeys, we integrated two scRNA-seq datasets from the rhesus monkey and a cynomolgus monkey using anchors and a canonical correlation analysis method. Single-cell data from the cynomolgus monkey were obtained from previously reported datasets^4^. After filtering, we analyzed 16,434 cells and identified 15,808 genes. Fourteen clusters were identified in the aggregated data using the nearest neighbor algorithm of Seurat analysis (Supplementary Fig. S2a). And then, ten cell subtypes were defined in the rhesus monkey (Fig. 2a) and eight cell subtypes in the cynomolgus monkey (Fig. 2b) based on UMAP coordinates and differential expression of specific marker genes between the identified clusters. Analysis of differentially expressed genes in each cluster revealed that eight cell subtypes were the same in both monkeys: naïve cells, Treg-, Tfh-, Th2-, Th17-, Eomes^+^ Tr1-, CTL-, and Eomes^+^ innate-like cells. A volcano plot (Fig. 2c) of the differentially expressed genes revealed that the rhesus monkey showed high expression of differentiation- or effector cell-associated genes such as *GZMB*, *KLRG1*, *KLRD1*, *MKI67*, *ITGB1*, *PDCD1*, and *CCR9*. By contrast, the cynomolgus monkey showed high expression of naïve- or regulatory T cell-associated genes such as *LEF1*, *TCF7*, *CCR7*, *PECAM1*, *IKZF2*, and *FOXP3*.

Most of the biomarker genes defining each cluster were similar in both monkeys; however, two cell subsets in the rhesus monkey differed from those in the cynomolgus monkey (Fig. 2d, e). First, a subset of CCR9^+^ Th-like cells that predominantly express *CCR9* was characterized by high expression of *ICAM2*, which plays a role in lymphocyte homing and recirculation^24^. The CCR9^+^ Th-like cells are distinguishable from Tfh-like cells due to low expression of *CXCR5* and *ICOS* (Fig. 2f), and also differ from Th17-like cells due to low expression of *RORC* and *CCR6* (Fig. 2g). Second, the gene signatures of cluster 10 of the rhesus monkey, which expressed *ZBTB16* exclusively, were distinct from those of the cynomolgus monkey. That is, we found that cluster 10 in the rhesus monkey expressed biomarker genes such as *KLRB1, KLRG1, MAP3K8, TYROBP,* and *ZBTB16,* which are known as innate-like markers^19,20^, along with high *CD8A* expression (Supplementary Fig. S2b). Furthermore, the gene signature of this cluster differs from that of Th17-like cells in that it shows high expression of *IL2RB*, *NCR3*, *TBX21*, *PRF1*, *TYROBP*, and *GZMB* (Fig. 2h). By contrast, the *ZBTB16*-expressing cell cluster (^##^cluster 10), which is a very small population in the cynomolgus monkey, showed the gene signature *RORC*, *CCR6*, *IL23R*, and *IL17RE,* with high expression of *CD8A* and *CD8B* (Fig. 2e,i). Thus, these cells are similar to a Th17-like cell subset rather than an innate-like cell subset in the cynomolgus monkey.

### Cell state transitions of circulating DP T cells

**Figure 3:**
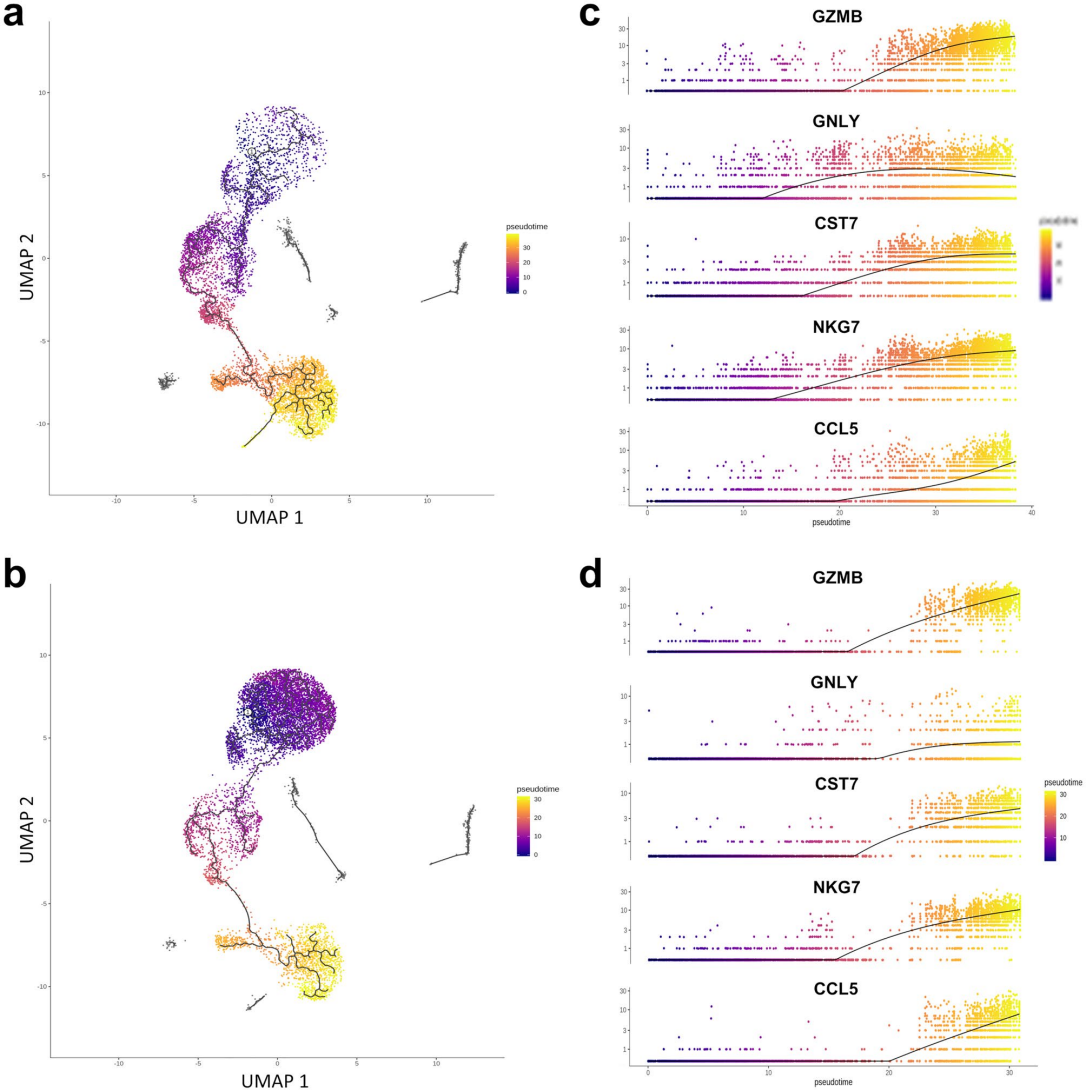
(**a, b**) UMAP visualizing the pseudotime trajectory of the aggregated dataset for DP T cells from (**a**) the rhesus and (**b**) a cynomolgus monkey. Black lines on the UMAP represent the trajectory graph. (**c, d**) Spline plots representing expression of genes with large transitions along the pseudotime in (**c**) the rhesus monkey and (**d**) the cynomolgus monkey. All plots were generated using Monocle3 0.1.2.

The cell state transitions of DP T cells were expressed as a trajectory graph based on the changes in the analyzed gene signature of DP T cells. To better understand the differentiation of DP T cells, we constructed single-cell trajectories using Monocle 3 (version 0.1.2); all aggregated data from DP T cells were assessed using these trajectories based on changes in the transcriptomes. Most DP T cells from the rhesus monkey were allocated to the mid-to-late pseudotime (Fig. 3a), whereas those from the cynomolgus monkey were allocated to an early pseudotime corresponding to naïve T cells (Fig. 3b). Interestingly, we found that CCR9^+^ Th-like cluster in the rhesus monkey formed a distinct branch in the trajectory map, despite their close relationship with neighboring Tfh- and Th17-like clusters. This branch of the CCR9^+^ cluster was not observed in the cynomolgus monkey, suggesting that CCR9^+^ Th-like cells are a specific subtype of circulating DP T cells in the rhesus monkey. Next, genes related to T cell differentiation whose expression changed significantly over pseudotime were selected. Most genes exhibiting a large transition in the rhesus monkey were cytotoxic-related genes such as *GZMB*, *GNLY*^17^, *CST7*^25^, *NKG7*^26^, and *CCL5*^27^ (Fig. 3c). A substantial number of single cells expressing these genes were allocated to the mid-to-late pseudotime; therefore, they differed from the transitions seen at a late pseudotime in the cynomolgus monkey (Fig. 3d). Given the expression patterns of these effector-related genes and their trajectory inferences, DP T cells from the rhesus monkey show a more diverse differentiation status than those from the cynomolgus monkey.

### Characteristics of the cell populations expressing ZBTB16

To better understand the characteristics of the cell subsets we identified, we next focused on how the distinct cell subsets are related. The number of clusters detected by the Louvain algorithm was 38 in the rhesus monkey (Supplementary Fig. S3a) and 44 in the cynomolgus monkey (Supplementary Fig. S3b). We found that many clusters in the rhesus monkey comprised mainly helper and cytotoxic cell populations, in contrast to those in the cynomolgus monkey that mostly comprised naïve cell populations. This implies that we are able to identify more diverse cell subtypes of DP T cells in the rhesus monkey than in the cynomolgus monkey.

**Figure 4:**
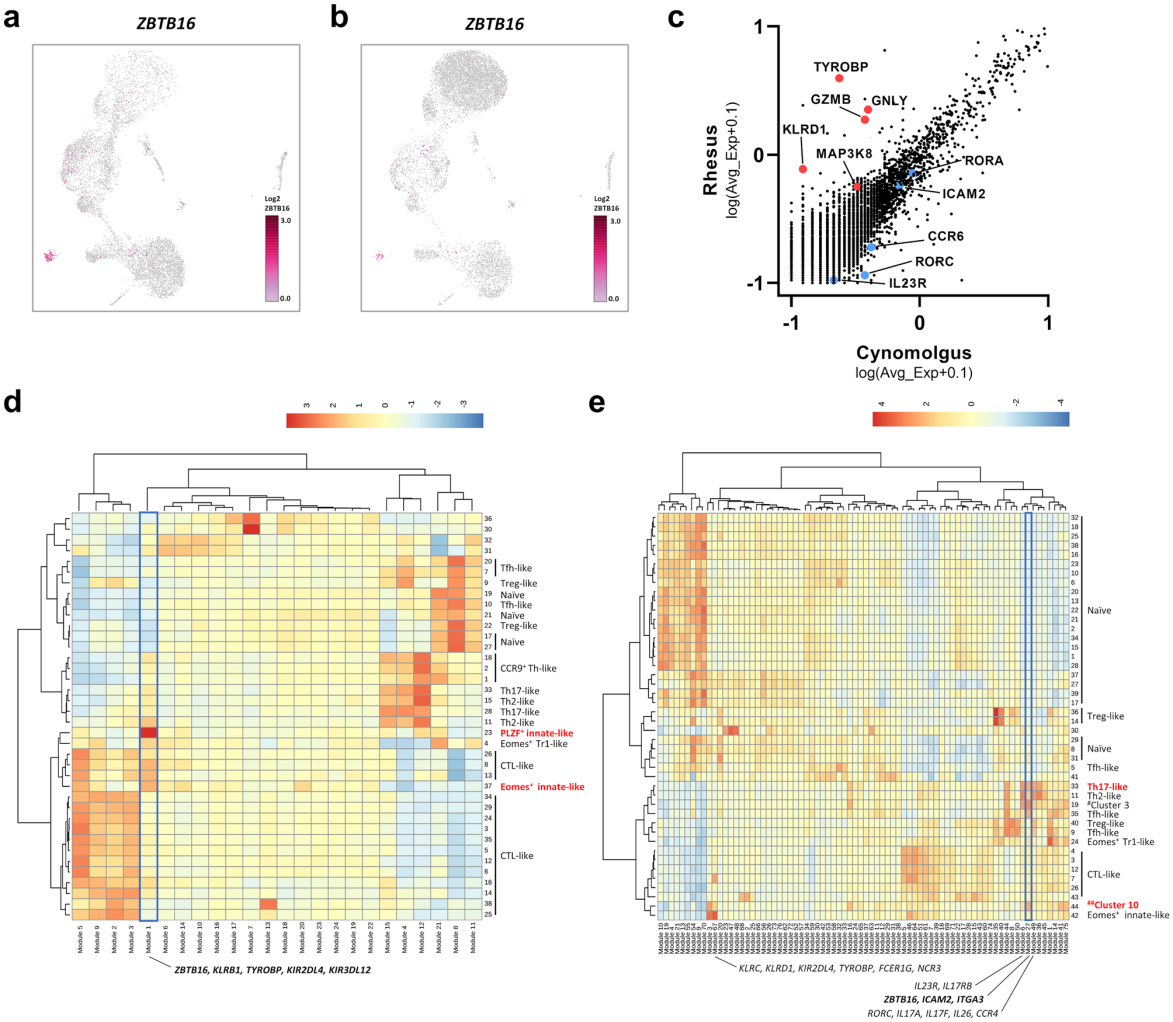
(**a, b**) UMAP representing the expression of *ZBTB16* (PLZF) in (**a**) the rhesus monkey and (**b**) the cynomolgus monkey. (**c**) Scatter plot showing differentially expressed genes between *ZBTB16*^+^ clusters from the rhesus and the cynomolgus monkey. (**d**) Heatmap showing aggregate expression of all genes in each module across all clusters detected in the rhesus monkey. (**e**) Heatmap showing aggregate expression of all genes in each module across all clusters detected in the cynomolgus monkey. The cluster number and the defined cell subset corresponding to each cluster are shown on the right side of the heatmap. Representative genes corresponding to the module are shown below the heatmap. Scatter plots were generated using GraphPad Prism 8.0.2. and heatmaps were generated using Monocle3 0.1.2.

Interestingly, clusters expressing *ZBTB16* showed sharply different characteristics between the two monkeys. That is, the cluster highly expressing *ZBTB16* in rhesus DP T cells is a well-defined subset and characterized by high expression of innate-like gene signatures such as *TYROBP*, *GZMB*, *GNLY*, *KLRD1*, and *MAP3K8* (Fig. 4a,c). In contrast, the *ZBTB16*^+^ cluster (^##^cluster 10) of cynomolgus DP T cells was a relatively small population, and exhibited high expression of Th17-associated gene signatures such as *RORC*, *RORA*, *CCR6*, and *IL23R* (Fig. 4b, c).

According to the co-regulated gene modules identified by Louvain community analysis, the *ZBTB16*-expressing cell population in the rhesus monkey clearly showed characteristics of innate-associated cells (Fig. 4d). That is, the cluster (23) expressing high *ZBTB16* levels also had a high expression score for module 1, which contains innate-related gene signatures such as *KLRB1*, *KIR2DL4*, *KIR3DL12*, and *TYROBP*. Furthermore, we found that two clusters (23 and 37) corresponding to PLZF^+^ innate-like cells and Eomes^+^ innate-like cells, respectively, were not only closely related to each other, but also exhibited much higher expression scores in module 1 than the other clusters. This was not the case in the cynomolgus monkey. In this monkey, the *ZBTB16*^+^ cell cluster (44) was closely related to the Eomes^+^ innate-like cell cluster (42), but the *ZBTB16*^+^ cell cluster exhibited low expression of module 67 associated with innate-like gene signatures (e.g., *KLRC*, *KLRD1*, *KIR2DL4*, *TYROBP*, *FCER1G*, and *NCR3*). Rather, the *ZBTB16*^+^ cell cluster showed high expression of module 27, including *ZBTB16*, *ICAM2*, and *ITGA3* (Fig. 4e), which is closely related to Th17-associated gene modules 6 and 49 (e.g., *RORC*, *IL17A*, and *IL23R*). Taken together, the data suggest that the PLZF^+^ innate-like cell subtype identified in the rhesus monkey showed gene signatures clearly distinct from those of other subsets with helper functions.

## Discussion

The data presented here demonstrate that circulating DP T cells from a rhesus monkey comprise heterogeneous populations with various characteristics. Compared with conventional single-positive T cells, DP T cells exhibit diverse features, including helper-, regulatory-, cytotoxic-, and innate-like roles. In this study, scRNA aggregation analysis based on scRNA-seq data from one rhesus and one cynomolgus monkey showed that the composition of circulating DP T cells in rhesus monkey is distinctly different from that in cynomolgus monkeys^4^. That is, we identified CCR9^+^ Th-like and PLZF^+^ innate-like cells in the rhesus monkey, two distinct subsets that were not revealed by previous scRNA-seq analysis of DP T cells from cynomolgus monkey.

CCR9^+^ Th cells, which comprise only a small fraction of CD4^+^ T cells in the lymphoid tissues and circulation of healthy mice and humans^28^, are a subset of T cells that migrate selectively to the gastrointestinal tract (GIT) and predominately express CCR9^29,30^. CCR9^+^ Th cells, which migrate to the GIT or pancreas via CCR9 and play an important role in inflammatory disease, show similar phenotypes to Tfh and Th17 cells and are often found at the site of inflammation as these cells^31^. That is, CCR9^+^ Th cells are similar to Tfh cells that produce IL-21, migrate to the GIT, and provide help to CD8^+^ T cells during the development of inflammatory diseases^31^. They also show characteristics similar to those of Th17 cells that secrete inflammatory cytokines such as IL-17, IL-21, and IL-22 and are involved in inflammatory diseases^28^. Thus, CCR9^+^ Th cells may contribute to the regional characteristics of organ-specific autoimmune diseases^31^. ICAM-2, which is highly expressed in this cluster, is readily detected on T and B cells from humans and rhesus monkeys^32^, and is implicated in lymphocyte homing and recirculation^24^. ICAM-2 may play a central role in the initial interaction between lymphocytes and antigen-presenting cells in that resting T lymphocytes express ICAM-2 but minimal levels of ICAM-1^33^. Thus, the CCR9^+^ Th-like cells we identified seem to play a crucial role in adaptive immunity associated with intestinal inflammation.

Additionally, identification of PLZF^+^ innate-like cells based on expression of *KLRG1*, *MAP3K8*, and *TYROBP* may reveal another type of unconventional T cell because these cells also express high levels of *KLRB1* and *ZBTB16*, both of which are hallmarks of innate lymphocytes^19,20^. A recent study reporting bulk scRNA-seq analysis of human thymus tissue showed that PLZF is highly expressed by a variety of unconventional T cells, including NKT-like cells and Th17-like cells^9^. However, the PLZF^+^ innate-like cell cluster we identified did not express the Th17-like cell-associated genes *RORC*, *CD40LG*, and *CCR6*, despite showing high expression of *ZBTB16* and *KLRB1*. Rather, given that PLZF^+^ innate-like cells in the rhesus monkey express high *IL2RB*, *IL15RA*, and CD8αα, this population may be similar to unconventional PLZF^+^CD8αα^+^ cells, which depend on IL-15 signaling for development and survival in human peripheral blood^34^. Indeed, this subset exhibits an activated/memory phenotype and are capable of controlling effector T cell responses via a perforin-dependent mechanism typical of innate-like cytotoxic cells^35^, rather than secretion of immunosuppressive cytokines (e.g., IL-10 and TGF-β)^34^. Previously, we showed that PLZF was expressed at higher levels by DP T cells than by conventional T cells in the peripheral blood and liver of rhesus monkeys^36^. Although their origin cannot yet be definitively determined, it seems that this population is distinctly related to innate-like cells given the innate-associated gene signatures identified from the scRNA-seq data.

Another difference between the DP T cells from the two monkeys is that they exhibit different cellular differentiation trajectories over pseudotime. Although the number of analyzed subjects is limited, we found that a large number of single cells from the rhesus monkey were allocated to the mid-to-late pseudotime, suggesting that DP T cells were more likely to transit into multipotential cell fates. By contrast, most cell clusters in the cynomolgus monkey were allocated to an early pseudotime, implying that they have not yet progressed to effector or differentiated status and remained naïve.

Taken together, despite the limitation of the small cell population, we were able to demonstrate the heterogeneity of DP T cells using scRNA-seq and trajectory inference. Although comparative analysis of aggregated scRNA-seq datasets from different individuals has allowed us to identify the characteristics of small cell subsets, it can be difficult to view these data as representing interspecies differences. Therefore, further bulk data analysis may be necessary. However, we believe that these findings will provide useful information to advance our understanding of the relationship between circulating DP T cells and the pathological process of immune-related diseases.

## Materials and methods

### Subjects

One healthy male rhesus monkey (*Macaca mulatta*) aged 6 years was used for the study. Blood samples for single-cell RNA-seq were obtained from the femoral vein. Single-cell RNA-seq analysis was performed on a sample from the same monkey. The animal was cared for in strict accordance with the National Institutes of Health Guide for the Care and Use of Laboratory Animals. The study was approved by the local Institutional Animal Care and Use Committee (IACUC) of Seoul National University Hospital (IACUC number:20–0004-S1A0). All experiments were performed in accordance with the relevant guidelines and regulations, and in accordance with the ARRIVE guidelines.

### Cell preparation for single-cell RNA-seq

Cell preparation for scRNA-seq was performed in the same manner as in our previous study^4^. Peripheral blood mononuclear cells (PBMCs) in heparin-anticoagulated whole blood were separated using the Ficoll density gradient method (GE Healthcare, Uppsala, Sweden). CD4^+^ T cells were sorted from fresh PBMCs using the REAlease CD4 MicroBead Kit (Miltenyi Biotec). Next, CD4^+^CD8^+^ DP T cells were sorted from MACS isolated CD4^+^ T cells using the CD8 Microbeads Kit (Miltenyi Biotec). The isolated CD4^+^CD8^+^ DP T cells were then labeled with the following Totalseq-CD4 and Totalseq-CD8 antibodies: CD4, Clone SK3, Barcode sequence: GAGGTTAGTGATGGA (BioLegend); CD8, Clone SK1, Barcode sequence: GCGCAACTTGATGAT (BioLegend). Isolated DP T cells were used for single-cell RNA seq-analysis, including library construction, sequencing, preprocessing, and advanced analysis, following the method in our previous study^4^.

### Library construction and sequencing

Libraries were prepared using the Chromium controller according to the 10 × Chromium Single-Cell V(D)J User Guide (10 × Genomics). Briefly, cell suspensions were diluted in nuclease-free water to achieve a targeted cell count of 10,000. The cell suspension was then mixed with master mix and loaded, along with Single-Cell 5′ Gel Beads and Partitioning Oil, into a Single-Cell A Chip. RNA transcripts from single cells were uniquely barcoded and reverse-transcribed within droplets. Next, cDNA molecules were pooled and enriched by PCR. For the 5′ Gene Expression Library, the cDNA pool went through an end repair process (i.e., addition of a single ‘A’ base), followed by ligation of the adapters. The products were then purified and enriched by PCR to create the 5′ Gene Expression Library. The purified libraries were quantified by qPCR according to the qPCR Quantification Protocol Guide (KAPA) and qualified using Agilent Technologies 4200 TapeStation (Agilent Technologies). Finally, the libraries were sequenced using the HiSeq platform (Illumina) according to the read length in the user guide.

### Preprocessing and analysis of single-cell RNA-Seq data

The Cell Ranger v3.1.0 (10 × Genomics) pipeline was used to generate FASTQ files from raw sequencing data for gene expression analysis of 5′ Gene Expression Library data, and for cell surface protein expression analysis of the FB library. Illumina basecall files for each sample, generated by the Illumina sequencing instrument, were converted to the FASTQ format using the ‘mkfastq’ command. Gene expression libraries were analyzed using the ‘count’ command. Sequence reads were aligned to the *Mmul_10* genome reference for *Macaca mulatta* using STAR (v2.5.1b) aligner. FB libraries were matched to the target FB reference. Next, gene and cell surface protein expression profiles for each cell were generated using information contained in the unique molecular identifier and 10 × cell barcode. Finally, cells were grouped into clusters according to gene expression.

### Advanced analysis of scRNA-Seq data

The raw count matrices of 10 × genomics were imported into Seurat 3.1.3. Raw counts for all genes expressed in >  = 3 cells, and of all cells with at least 200 detected genes, were used for downstream analysis. Since there may be a rare subset of cells with a clear outlier number of genes detected as potential multiplets, and there may be low-quality or dying cells, cells with over 4000 genes or mitochondrial counts over 20% were filtered out. After filtering, the count value of 14,459 genes across 7601 cells was normalized and scaled. Clustering and UMAP analysis were performed based on statistically significant principal components. Next, markers significant for each cluster compared with all remaining cells were identified using the minimum fraction of min.pct cells and the Wilcox rank sum test, reported only the positive ones. Gene-Enrichment and Functional Annotation analysis of the significant probe list was performed using the g:Profiler tool (https://biit.cs.ut.ee/gprofiler/). All data analysis using Seurat 3.1.3 was performed with standard settings.

### Aggregation and trajectory analysis of scRNA-seq data

As with data from the rhesus monkey, published data for cynomolgus monkeys were preprocessed in the same way as in the previous study^4^. The resulting data were aggregated with data of the rhesus monkey into a single dataset to analyze samples using the Cell Ranger ‘aggr’ command. Raw count matrices aggregated by Cell Ranger ‘aggr’ were also processed as described above; after filtering, 15,808 genes across 16,434 cells were selected for analysis in the next step. Cell pairwise anchor correspondences between different single-cell data sets were identified with 15-dimensional spaces by canonical correlation analysis. Using these anchors, two datasets were integrated and transformed into a shared space. Gene expression values for each gene were scaled and normalized across all integrated cells. Clustering and UMAP analysis were performed as mentioned above. Additionally, rhesus monkey and cynomolgus monkey data within each cluster were compared using the Wilcox rank sum test (min.pct = 0.1; logfc.threshold = 0.4054651; min.cells.group = 3; min.cells.feature = 3; positive/negative ones were filtered) to identify differentially expressed genes. Gene-Enrichment and Functional Annotation analysis of significant gene lists from the two datasets was performed using g:Profiler tool (https://biit.cs.ut.ee/gprofiler/).

Trajectory reconstruction for each data set was performed by Monocle3 0.1.2, which defines a pseudotime measurement across a root cluster group through which the dynamics of gene expression can be examined.

## Supplementary Information

Supplementary Information.

## Data Availability

All scRNA-seq data are deposited in the Gene Expression Omnibus (GEO) accession number GSE199564.
