# Supplementary material for: Heterogeneity of circulating CD4+CD8+ double-positive T cells characterized by scRNA-seq analysis and trajectory inference
Source: Sci Rep. 2022 Aug 18;12:14111. doi: 10.1038/s41598-022-18340-3 (PMC9388645; doi:10.1038/s41598-022-18340-3)
Supplement: Supplementary file 1 — Supplementary Information. [file 41598_2022_18340_MOESM1_ESM.pdf]

Heterogeneity of circulating CD4+CD8+ double-positive T cells characterized by scRNA-seq analysis and trajectory inference

Sung Min Choi[1], Hi Jung Park[1], Eun A Choi[1], Kyeong Cheon Jung[2,3,4], and Jae Il Lee[2,5,*]

Supplementary Figure

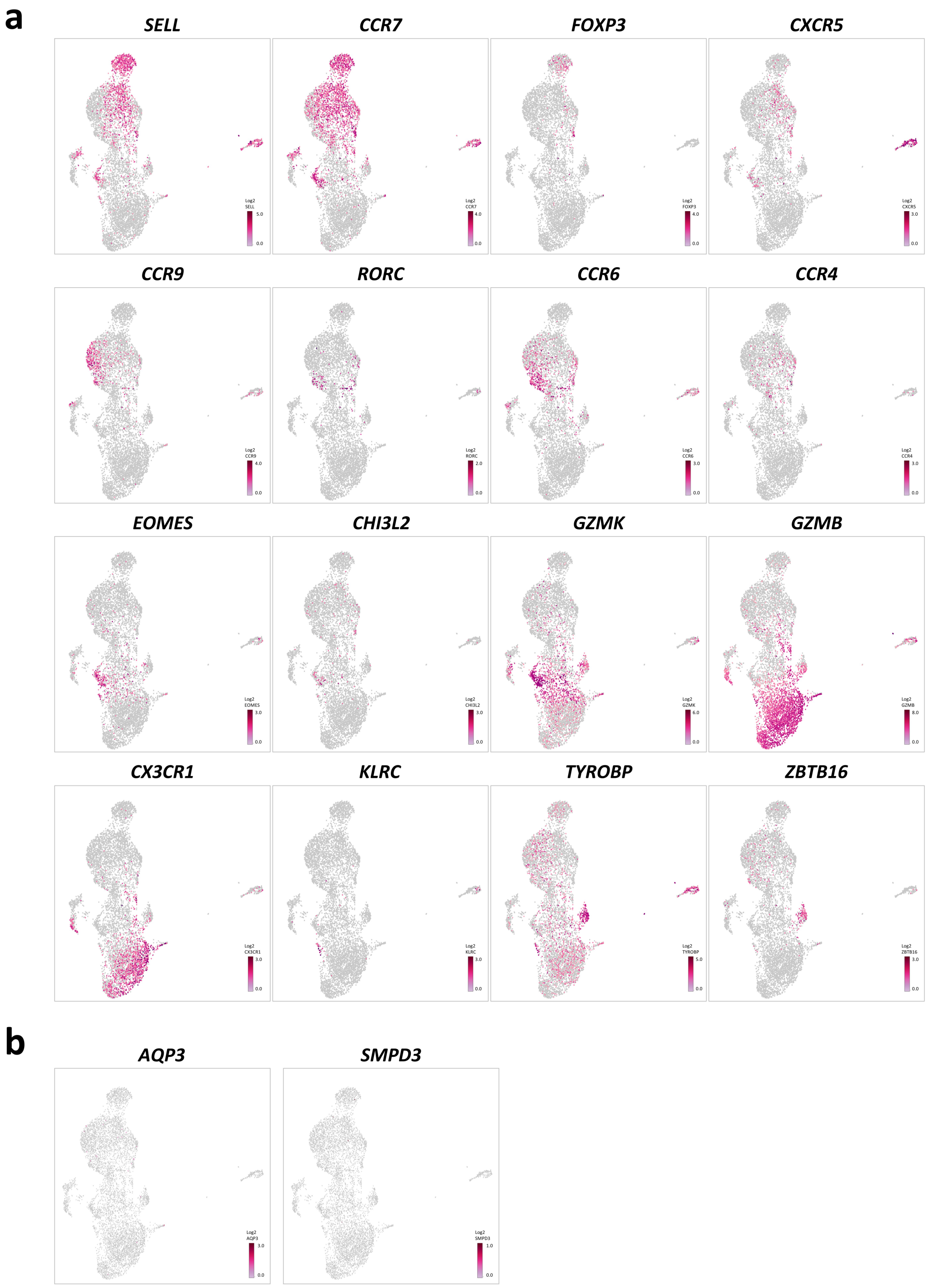

Supplementary Figure 1. (**a**) UMAP representing expression of marker genes: *SELL*, *CCR7*, *FOXP3*, *CXCR5*, *CCR9*, *RORC*, *CCR6*, *CCR4*, *EOMES, CHI3L2*, *GZMK*, *GZMB*, *CX3CR1*, *KLRC*, *TYROBP*, and *ZBTB16* in rhesus monkey. (**b**) UMAP representing expression of *AQP3* and *SMPD3* in rhesus monkey.

Supplementary Figure

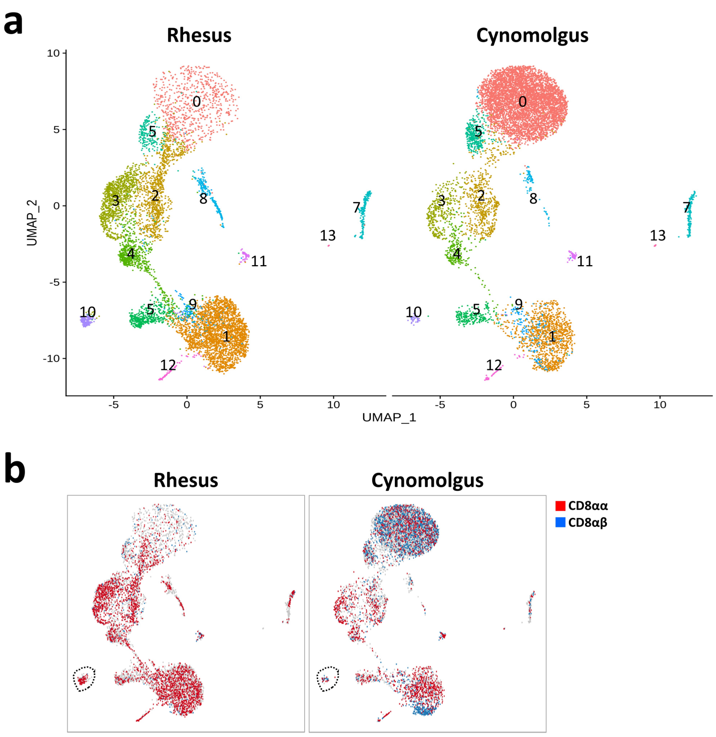

Supplementary Figure 2. (**a**) UMAP representing clusters of DP T cells from the rhesus (left) and a cynomolgus monkey (right) identified using the nearest neighbor algorithm in the Seurat analysis platform. The number in UMAP indicates the cluster number. (**b**) UMAP representing expression of CD8αα and CD8αβ. For PLZF^{+} cluster(dotted line), cells expressing *CD8A* alone (CD8αα^{+}) are dominant in rhesus monkey (left). On the other hand, the levels of cells expressing *CD8A* alone (CD8αα^{+}) and cells expressing *CD8B* (CD8αβ^{+}) are similar in cynomolgus monkey (right).

Supplementary Figure

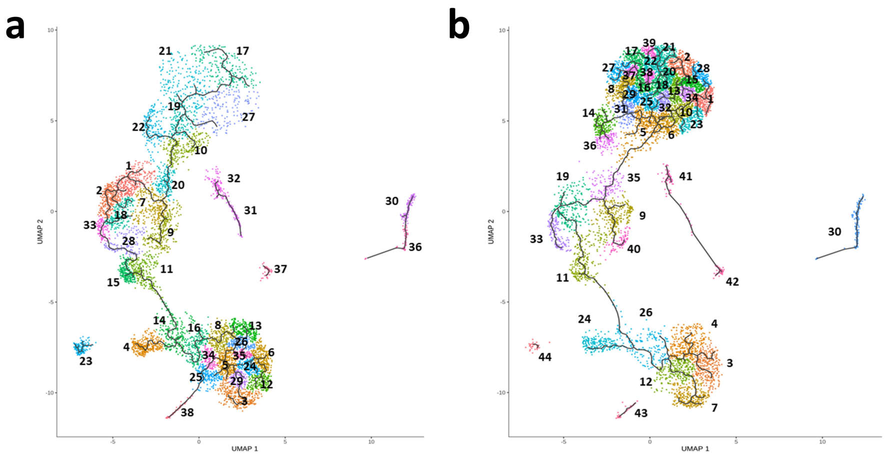

Supplementary Figure 3. UMAP representing clusters of (**a**) the Rhesus and (**b**) a cynomolgus DP T cells detected by Louvain's algorithm. Black lines on the UMAP represent the trajectory graph. The number in UMAP indicates the cluster number.
